# Diversification and subspecies patterning of the goitered gazelle (*Gazella subgutturosa*) in Iran

**DOI:** 10.1002/ece3.6324

**Published:** 2020-05-08

**Authors:** Davoud Fadakar, Eva V. Bärmann, Hannes Lerp, Masoumeh Mirzakhah, Maryam Naseri Nasari, Hamid Reza Rezaei

**Affiliations:** ^1^ Department of Natural Resources Isfahan University of Technology Isfahan Iran; ^2^ Department of Fishery and Environment Gorgan University of Agricultural Science and Natural Resources Gorgan Iran; ^3^ Zoological Research Museum Alexander Koenig Bonn Germany; ^4^ Natural History Collections Wiesbaden Germany

**Keywords:** conservation genetics, cytochrome *b*, desert ungulate, geographic barriers, haplotype network, molecular phylogeny

## Abstract

Goitered gazelles, *Gazella subgutturosa,* exist in arid and semiarid regions of Asia from the Middle to the Far East. Although large populations were present over a vast area until recently, a decline of the population as a result of hunting, poaching, and habitat loss led to the IUCN classification of *G. subgutturosa* as “vulnerable." We examined genetic diversity, structure, and phylogeny of *G. subgutturosa* using mitochondrial cytochrome *b* sequences from 18 geographically distant populations in Iran. The median‐joining network of cyt *b* haplotypes indicated that three clades of goitered gazelles can be distinguished: a Middle Eastern clade west of the Zagros Mountains (and connected to populations in Turkey and Iraq), a Central Iranian clade (with connection to Azerbaijan), and an Asiatic clade in northeastern Iran (with connection to Turkmenistan, Uzbekistan, and other Asian countries as far as northeastern China and Mongolia). Based on our results, we argue that Iran is the center of diversification of goitered gazelles, due to the presence of large mountain ranges and deserts that lead to the separation of populations. In accordance with previous morphological studies, we identified the Asiatic clade as the subspecies *G. s. yarkandensis*, and the other two clades as the nominate form *G. s. subgutturosa*. The new genetic information for goitered gazelles in Iran provides the basis for future national conservation programs of this species.

## Graphical abstract

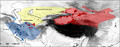

## 1 INTRODUCTION

It is generally accepted that maintaining genetic diversity is necessary for species conservation (Reed & Frankham, 2003). Thus, clarifying the evolutionary relationships between populations or even geographic subspecies is pivotal, before conservation measures will be implemented (Avise, 1989). One species for which such data are not available across its range is the goitered gazelle (*Gazella subgutturosa*), a wide‐ranging ungulate that occurs east of the Tigris/Euphrates Basin to eastern Turkey, north into the Caucasus and across Iran into Turkmenistan, Uzbekistan, Kazakhstan, and from there into northern China and Mongolia (Groves, 1985; Kingswood & Blank, 1996; Mallon & Kingswood, 2001; Mirzakhah, Naderi, Rezaei, Fadakar, & Naseri, 2015). Despite the fact that morphology, taxonomy, and ecology of this species were the focus of several studies (Fakheran Esfahani & Karami, 2005; Farhadinia et al., 2009; Hayatgheib, Karami, Farahmand, Mehrabani‐yeganeh, & Farhadinia, 2011; Hemami, 1994; Hemami & Groves, 2001; Karami, Hemami, & Groves, 2002; Makki, Fakheran, Moradi, Iravani, & Senn, 2013; Nowzari, Hemami, & Behrouzi Rad, 2007), information on their genetic relationships is scarce in Iran (Fadakar et al., 2013), and most Iranian populations were not yet studied with regard to phylogeny and population genetic structure (but see Fadakar et al., 2013; Khosravi, Malekian, Hemami, Silva, & Brito, 2019; Mirzakhah et al., 2015; Zachos et al., 2010).

### 1.1 Taxonomy of goitered gazelles

**Figure 1:**
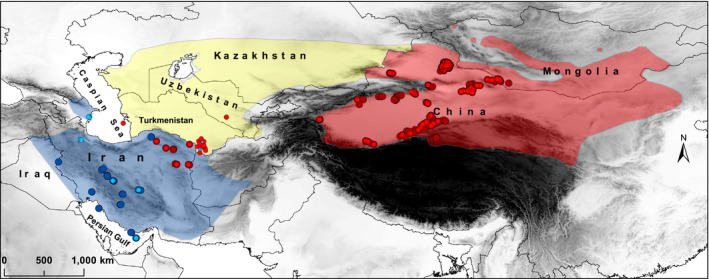
Distribution of subspecies of *G. subgutturosa* in Asia based on the morphological data, cyt *b* sequences, and D‐loop sequences. Blue (*G. s. subgutturosa*)*,* yellow (*G. s. gracilicornis*), and red (*G. s. yarkandensis*) polygons correspond to the distribution based on the morphological data (Groves & Grubb, 2011) which are modified from distribution map of *G. subgutturosa* (IUCN SSC Antelope Specialist Group, 2017), large circles represent samples sequenced for cyt *b*, small circles represent samples sequenced for D‐loop. Genetically identified subspecies (based on this study) are represented with blue (*G. s. subgutturosa*) and red (*G. s. yarkandensis*) circles. The locations of sequences of China and Mongolia are approximate. Different shades of gray represent different altitudes

In recent taxonomic literature, following the revision by Groves and Grubb (2011), some of the previously recognized subspecies of goitered gazelle were elevated to full species status based on morphological data: the Yarkand gazelle (*G. yarkandensis*) in China and Mongolia (including the previously recognized subspecies *G. s. hillieriana*, *G. s. sairensis*, *G. s. reginae*, and *G. s. mongolica*), the Turkmen goitered gazelle (*G. gracilicornis*) in Turkmenistan, Kazakhstan, Tajikistan, and Uzbekistan, and the nominate subspecies, the Persian gazelle (*G. subgutturosa*) in Iran (including a previously recognized subspecies, the Seistan gazelle, *G. s. seistanica*, from eastern Iran), Iraq, Azerbaijan, and Turkey (Figure 1). Recent molecular studies show that differences between these species are only detectable when using fast‐evolving mitochondrial markers, preferably control region sequences (e.g., Abduriyim, Zibibulla, Eli, Ismayil, & Halik, 2018b), so a distinction on species level might not be appropriate, and the groups are referred to as subspecies throughout this manuscript. However, molecular data were able to support subspecific patterning within *G. subgutturosa*. Sorokin, Soldatova, Lukarevskiy, and Kholodova (2011) found a marked distinctness of *G. s. subgutturosa* from Azerbaijan compared to *G. s. gracilicornis* from Turkmenistan and Uzbekistan. Abduriyim, Zibibulla, et al. (2018) distinguished three subspecies in Asia: *G. s. gracilicornis* (referred to as *G. s. subgutturosa* in their study, but sequences from Iran and Azerbaijan were hardly included) stretching from Turkmenistan to eastern Kazakhstan, *G. s. hillieriana* (possibly including *G. s. reginae* and *G. s. sairensis*) in northern China and Mongolia, and *G. s. yarkandensis* in southern Xinjiang Uygur Autonomous Region (XUAR) in China. The majority of the recent studies, however, focused on population genetics of specific regions within the range of *G. subgutturosa*, for example XUAR in China (Abduriyim, Nabi, & Halik, 2018a; Abduriyim, Zibibulla, et al., 2018; Dong et al., 2016) or Iran (Fadakar et al., 2019; Khosravi et al., 2019; Mirzakhah et al., 2015; Zachos et al., 2010) without addressing taxonomic questions.

### 1.2 Conservation and population status of goitered gazelles in Iran

Knowing more about the population genetics of *G. subgutturosa* in Iran is important to improve their conservation. The species is associated with open plains near hilly escape terrain that decreases their susceptibility to poachers (Farhadinia et al., 2009). It is widely distributed in all steppes or semideserts in Iran, except in the far northwest, along the Caspian Sea, and in the southeast (Firouz, 2005; Karami et al., 2002). Even though many areas exist with high numbers of resident *G. subgutturosa*, for example, Mooteh Wildlife Refuge (>7,000) and Qamishlou National Park (>3,000) in Isfahan Province, populations have declined considerably during the last four decades (Hemami & Groves, 2001; Karami et al., 2002). The species has even disappeared from some protected areas, for example, Borouieh Wildlife Refuge (WR) in Central Iran (Fadakar et al., 2013), as well as many nonprotected areas, for example, the Chenaran plain of Razavi Khorasan Province and Turkmen Sahra of Golestan Province in northeastern Iran.

In the early 1990s, about 100,000 individuals of *G. subgutturosa* occurred in Asia, but now the species is threatened in many parts of its natural range (Mallon & Kingswood, 2001), which led to the IUCN classification “vulnerable” in 2008 (IUCN SSC Antelope Specialist Group, 2017). Hunting and habitat loss have caused a recent decline of more than 30% in many populations (IUCN SSC Antelope Specialist Group, 2017). In Iran, intensified hunting and habitat destruction due to overgrazing, conversion of natural gazelle habitat to agricultural land, urbanization, road development, and mining account for the dramatic decline of *G. subgutturosa* in deserts and plains (Fadakar et al., 2013, 2019; Karami et al., 2002; Mallon & Kingswood, 2001; Mirzakhah et al., 2015).

One recent study showed that a reintroduced population of *G. subgutturosa* in Dimeh Protected Area (PA) is actually a mixed population of *G. subgutturosa* and *G. marica* (Fadakar et al., 2019). These were considered to be conspecific based on external appearance and translocated from two source populations without prior knowledge on their genetic identity. Also, reintroduced goitered gazelle individuals from eastern Turkey to eastern Georgia showed haplotype identity of *G. marica* (Murtskhvaladze, Gurielidze, Kopaliani, & Tarkhnishvili, 2012). These illustrate how important it is to analyze genetic markers before carrying out further translocations of gazelles, especially in Iran where three species, *G. subgutturosa*, *G. bennettii*, and *G. marica*, occur partially in sympatry (Groves, 1997; Groves & Harrison, 1967), that is, the geographic ranges of *G. marica* and *G. subgutturosa* overlap in southwestern Iran, and the geographic ranges of *G. subgutturosa* and *G. bennettii* meet in Yazd Province, Central Iran, where both species occur in neighboring areas (*G. subgutturosa* in Kalmand‐Bahadoran PA, *G. bennettii* in Darre Anjir WR). Also, a resident population of *G. marica* in Mond PA is in close proximity to the habitat of *G. bennettii* in Nayband National Park (NP) northwest of the Persian Gulf.

Another important aspect for the *in situ* conservation of *G. subgutturosa* in Iran is to understand the connectivity of (sub‐) populations for assessing the impact of inbreeding, or for evaluating which areas are most important for maintaining a healthy population. The Zagros mountain range that stretches through the country from the northwest to the southeast separates suitable gazelle habitats, and significant morphological differences between gazelle populations east and west of the mountain range are detectable (Hayatgheib et al., 2011). Contrastingly, in the central Iranian Plateau large groups of *G. subgutturosa* exist, that migrate between protected areas (Khosravi et al., 2018), for example, from Mooteh WR to Qamishlou NP in winter, and back to the Mooteh WR in summer (Fakheran Esfahani & Karami, 2005) and from Mooteh WR to northern areas such as Haftad Gholle PA. Therefore, genetic connectivity can be assumed between those populations.

With our study, we want to broaden the taxonomic picture for *G. subgutturosa* by sequencing the complete cytochrome *b* (cyt *b*) gene for this species in Iran. The new sequences are combined with published cyt *b* sequences of *G. subgutturosa* from Asia (Abduriyim, Nabi, et al., 2018; Dong et al., 2016) and other published mitochondrial sequences available on GenBank to study the phylogeny and subspecific patterning of *G. subgutturosa*. We aim to infer for the first time a genetic framework for Iranian *G. subgutturosa* populations living in almost all parts of the country, in order to understand the phylogenetic relationships among them, and with *G. subgutturosa* in other parts of Asia. Therefore, we analyzed the mitochondrial sequence variation of cyt *b* from 18 sampling sites. We hypothesized that (1) all supposed Iranian *G. subgutturosa* populations belong to this species and no further maternal introgression from *G. marica* occurred, and (2) the Zagros mountain range acts as a geographical barrier between the gazelle populations that occur east and west of the mountains, as was proposed based on morphological studies (Geptner, Nasimovich, & Bannikov, 1961; Hayatgheib et al., 2011).

## 2 MATERIAL AND METHODS

### 2.1 Sampling

**Figure 2:**
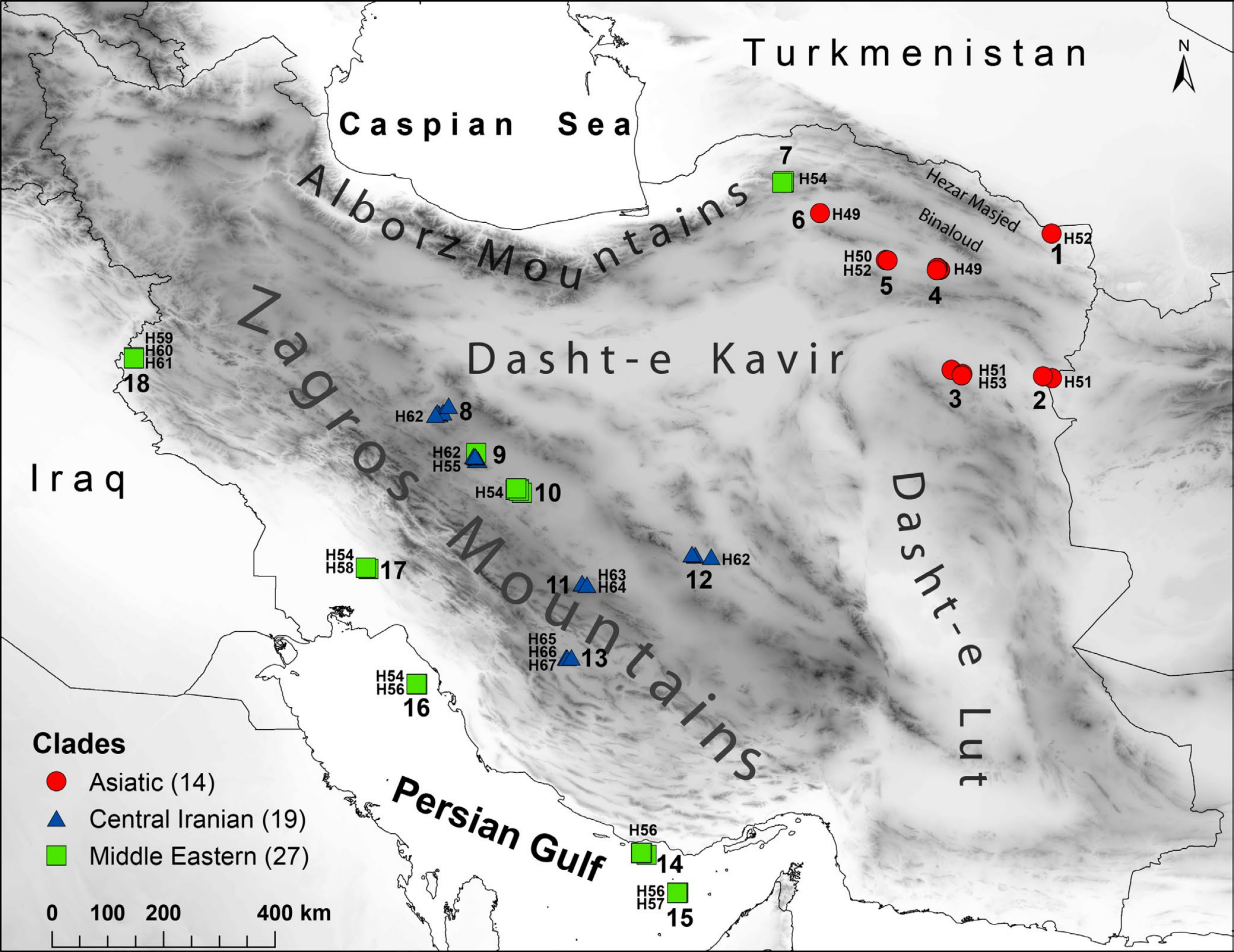
Sample locations of *G. subgutturosa* in Iran: 1 = Jangal‐e Khajeh PA (KHJE), 2 = Khaf Breeding Center (KHAF), 3 = Hengam PA (HNGM), 4 = Reisi PA (REIS), 5 = Shirahmad WR (SHIR), 6 = Miandasht WR (MNDT), 7 = Golestan NP (GLSN), 8 = Mooteh WR (MOTE), 9 = Qamishlou NP (QMIS), 10 = Kolah Ghazi NP (KOLA), 11 = Abadeh PA (ABAD), 12 = Kalmand‐Bahadoran PA (KALM), 13 = Bamu NP (BAMU), 14 = Kish Island (KISH), 15 = Siri Island (SIRI), 16 = Kharg Island (KHRG), 17 = Dimeh PA (DIME), 18 = Qaraviz Hunting Prohibited Area (QRVZ). Each specimen is represented by an icon according to its haplotype clade, red circle = Asiatic clade, blue triangle = Central Iranian clade, green square = Middle Eastern clade, and the haplotype number according to Figure 3 is given next to the icon. The background hillshade was made using the Shuttle Radar Topography Mission (SRTM) elevation model (http://srtm.csi.cgiar.org) in QGIS version 3.10; country boundaries were downloaded from DIVA‐GIS dataset (http://www.diva‐gis.org/Data)

This study was conducted with permission by the Iranian Department of Environment that authorized access to all sampling locations. In total, 60 fecal samples from *G. subgutturosa* were collected from 18 different localities in Iran (Figure 2). Information regarding the origin, collector, and kind of material are summarized in Table S1 for each sample (Supporting Information). Fresh feces were collected in the field, after observing the animals from a distance to allow for species identification. Samples were stored in 96% ethanol.

### 2.2 DNA extraction, amplification, and sequencing

Whole genomic DNA was extracted from fecal samples using AccuPrep genomic DNA extraction tissue kit (Bioneer) following the manufacturer's instructions. Polymerase chain reaction (PCR) was performed for amplification of the complete coding region of the cyt *b* gene of the mitochondrial genome using the primers L14724: 5′‐TGA CTA ATG ATA TGA AAA ACC ATC GTT G‐3′ and H15915: 5′‐TGC TCT CCT TCT CTG GTT TAC AAG AC‐3′ (Lerp, Wronski, Pfenninger, & Plath, 2011). In case of amplification failure, CYTB_F (5′‐CCCCACAAAACCTATCACAAA‐3′) and CYTB_R (5′‐AGGGAGGTTGGTTGTTCTCC‐3′) primers (Pedrosa et al., 2005; Rezaei et al., 2010) were used.

The reaction mixture was prepared in 25 μl volume, containing 1 unit of Euro Taq DNA polymerase, 10 µM Tris‐HCl, 30 µM KCl, 1.5 mM MgCl_2_, 250 µM of each dNTP, and 2 pmol primers (Bioneer, South Korea).

The thermocycling conditions for L14724 and H15915 primers was performed as follows: initial denaturation (3 min at 95°C), followed by five cycle steps of 60 s at 94°C (denaturation), 90 s at 45°C (primer annealing) and 90 s at 72°C (elongation), then 40 cycle steps of 60 s at 94°C, 60 s at 50°C and 90 s at 72°C, and lastly, a final extension step (10 min at 72°C) (Lerp et al., 2011). For CYTB_F and CYTB_R primers, we used the following protocol (Rezaei et al., 2010): 10 min at 95°C followed by 35 cycles of 30 s at 95°C, 30 s at 55°C, and 60 s at 72°C, and finally followed by 7 min at 72°C.

Sanger sequencing was performed using the BigDye Terminator Cycle Sequencing kit v.3.1 (Applied BioSystems), and electrophoresis of the purified sequencing product was carried out on an ABI PRISM 3730xl automatic sequencer.

Sequences were edited for correction with SeqScape v.2.6 (Applied Biosystems). All new sequences have been submitted to GenBank (accession numbers: MT264037‐MT264088, Table S1, Supporting Information).

### 2.3 Alignments

For subsequent analyses, we worked with different alignments. Alignment 1 is restricted to the 60 new Iranian sequences generated in this study (i.e., “Western group” in Tables 1 and 2). Alignment 2 (i.e., “Total group” in Tables 1 and 2) additionally includes 219 cyt *b* sequences (48 haplotypes) referred to as *G. subgutturosa* from GenBank (see Table S1 for accession numbers). The majority of these (46 haplotypes) came from Xinjiang Uygur Autonomous Region (XUAR) in northwestern China (KM978960‐KM978991 and LC333582‐LC333595), and one from Mongolia (KU560652). All these additional Asian samples are referred to as “Eastern group” in Tables 1 and 2. The origins of the other three sequences (AF036282, NC020710, and JN632644, all have the same haplotype) are unknown. Furthermore, we used eight sequences from other closely related *Gazella* species as outgroup representatives [sand gazelle (*G. marica*), chinkara (*G. bennettii*), Cuvier's gazelle (*G. cuvieri*), and slender‐horned gazelle (*G. leptoceros*); Table S1]. The final dataset of complete cyt *b* (Alignment 2) comprises 279 sequences of *G. subgutturosa* and 8 outgroup sequences. Alignment 3 further includes 32 partial cyt *b* sequences of *G. subgutturosa* and is therefore restricted to only 390 bp. The additional sequences come from our study (in cases where no complete cyt *b* fragment could be obtained) and from GenBank, restricted to samples with known geographic provenance (Fadakar et al., 2013; Khosravi et al., 2019; Mirzakhah et al., 2015). Sequences were aligned using the Clustal W algorithm (Thompson, Higgins, & Gibson, 1994) implemented in Mega v.5 (Tamura et al., 2011), and obvious misalignments were corrected by eye.

**Table 1:** Cyt *b* mtDNA genetic diversity revealed for several subgroups of *G. subgutturosa*

Geographic groups and phylogenetic clades	*n*	S	H	*h* ± *SD*	*π* ± *SD*	*k*
Eastern group (China + Mongolia)	216	61	47	0.858 ± 0.019	0.00198 ± 0.00012	2.25431
Western group (Iran)	60	25	19	0.873 ± 0.015	0.00287 ± 0.00017	3.26625
Total group (Western + Eastern+unknown)	279	85	67	0.909 ± 0.012	0.00250 ± 0.00012	2.85434
Asiatic clade (*G. s. yarkandensis*)	233	67	53	0.877 ± 0.017	0.00215 ± 0.00012	2.45005
Central Iranian clade	19	8	6	0.468 ± 0.140	0.00090 ± 0.00042	1.02924
Middle Eastern clade	27	9	8	0.715 ± 0.061	0.00114 ± 0.00026	1.30484
Middle Eastern clade + Central Iranian clade (*G. s. subgutturosa*)	46	18	14	0.815 ± 0.033	0.002 ± 0.00024	2.27923

Note

*n* = number of individuals; S = number of segregating sites, H = number of haplotypes; *h* = haplotype diversity; *π* = nucleotide diversity; *k* = mean number of pairwise differences.

**Table 2:** Tests for population expansion for several subgroups of *G. subgutturosa* using *R_2_* (Ramos‐Onsins & Rozas, 2002), Tajima's *D* (Tajima, 1989), and Fu's *F*_s_ (Fu, 1997)

Geographic groups and phylogenetic clades	*R_2_*	*p*	Tajima's *D*	*p*	Fu's *F*_s_	*p*
Eastern group (China + Mongolia)	0.0197	**	−2.3758	**	−48.319	**
Western group (Iran)	0.0626	n.s.	−1.2387	n.s.	−6.608	*
Total group (Eastern + Western+unknown)	0.0167	**	−2.3963	**	−76.860	**
Asiatic clade (*G. s. yarkandensis*)	0.0190	**	−2.3704	**	−56.638	**
Central Iranian clade	0.1177	n.s.	−1.8824	**	−2.262	*
Middle Eastern clade	0.0846	n.s.	−1.405	n.s.	−3.084	*
Middle Eastern clade + Central Iranian clade (*G. s. subgutturosa*)	0.0647	n.s.	−1.4146	n.s.	−5.269	*

Note

n.s. = not significant.

* Significant (*p* < .05);

** Highly significant (*p* < .01).

### 2.4 Haplotype network

Based on Alignment 1, a median‐joining (MJ) network was constructed for all 60 sequences from Iran using PopART v.1.7 (Leigh & Bryant, 2015) with the default settings. A second MJ network was generated, using 279 sequences (Alignment 2), including 60 sample sequences from Iran (i.e., Alignment 1 with 19 Haplotypes) plus 219 published sequences (48 haplotypes) for whole Asia. For this haplotype network, we had to work around the problem that 13 sequences (from Abduriyim, Nabi, et al., 2018) were only 995 bp in length. As we did not want to lose the information from the remaining 145 bp that show some mutations mostly in Iranian samples, we filled in the missing information for the shorter sequences using the consensus sequence of all full‐length Asian samples (from Dong et al., 2016). Only three of these 32 sequences showed one unique single point mutation each in these parts of the sequence, so the consensus most likely reflects the true sequence information of the shorter sequences. The same was done for the sequence from Mongolia (Lerp et al., 2016) that has a length of 1,083 bp.

### 2.5 Phylogenetic analysis

Alignment 2 was used for a phylogenetic analysis of *G. subgutturosa*. Each haplotype was represented by a single specimen to avoid redundancy. The best‐fitting partitioning scheme and nucleotide substitution models were estimated using greedy search algorithm with PhyML (Guindon et al., 2010) in PartitionFinder v.2.1.1 (Lanfear, Calcott, Ho, & Guindon, 2012; Lanfear, Frandsen, Wright, Senfeld, & Calcott, 2016). We tested among partitioning schemes including division of protein‐coding genes into 1st, 2nd, and 3rd codon positions. Models were selected by the Bayesian information criterion (BIC). We found the optimal partitioning scheme includes three partitions (optimal models are shown in brackets) 1st codon (K80), 2nd codon (HKY + I), and 3rd codon (GTR + G). Phylogenetic analysis using Bayesian inference was carried out in MrBayes v.3.2.7a (Ronquist et al., 2012) with two independent runs of four Markov chains (one cold and three heated) over 10,000,000 generations and sampling every 1,000 generations. The first 25% of the sampled trees and estimated parameters were discarded as burn‐in. Convergence onto the stationary distribution was monitored with average standard deviation of split frequencies (below 0.01), the potential scale reduction factor (close to 1 for all parameters) in MrBayes v.3.2.7a (Ronquist et al., 2012), and the effective sample size (ESS) value (above 200) in Tracer v.1.7.1 (Rambaut, Drummond, Xie, Baele, & Suchard, 2018). The consensus phylogenetic tree was then edited in FigTree v.1.4.4 (http://tree.bio.ed.ac.uk/software/figtree/).

### 2.6 Population differentiation and structure

Measures of DNA polymorphism were estimated using DnaSP v.5 (Librado & Rozas, 2009) for several geographic and phylogenetic groups, that is, Western group (samples from Iran), Eastern group (samples from China and Mongolia), Total group, Central Iranian clade, Middle Eastern clade, and Asiatic clade. These include haplotype diversity (*h*, the probability that any two randomly sampled haplotypes are different—(Nei, 1987)), nucleotide diversity (*π*, the average number of nucleotide differences per site), and the mean number of pairwise differences within a group (*k*).

We calculated mismatch distributions separately for the phylogenetic subgroups and the total group to test if their frequency graph shows a chaotic/multimodal pattern characteristic for populations in demographic equilibrium, or a unimodal profile which is found in populations that experienced recent geographic expansion (Hey & Nielsen, 2004). The test was performed using Arlequin v.3.5.2.2 (Excoffier, Laval, & Schneider, 2005) under the null hypothesis that the observed data fit the sudden expansion model, and their fit to Poisson distributions was assessed by 1,000 Monte Carlo random simulations.

Several other statistics are frequently used in the literature for analyzing population expansions or declines, for example, *R*_2_ (Ramos‐onsins & Rozas, 2006), and Tajima's *D* (Tajima, 1989) which use information on the frequency of segregating sites, or Fu's *F*_s_ (Fu, 1997) which is a measure for estimating the amount of rare alleles (young mutations in expanding populations), where a negative value is interpreted as indicating recent demographic expansion. We applied these statistics to our dataset using the program DnaSP v.5 (Librado & Rozas, 2009).

### 2.7 Additional sequencing of mitochondrial D‐loop sequences

During the course of this study, we found it quite dissatisfying that for some already sampled populations of *G. subgutturosa*, especially in Azerbaijan, Turkmenistan, and Uzbekistan, cyt *b* sequences were not available. Restricting the study to this marker would limit the analysis of subspecies patterning of *G. subgutturosa*, as potentially an entire subspecies— *G. s. gracilicornis*—would be left out. We therefore decided to sequence ten of our samples for mitochondrial D‐loop, as this marker has been used in other studies of *G. subgutturosa* (Abduriyim, Zibibulla, et al., 2018; Sorokin et al., 2011; Zachos et al., 2010) and sequences are available on GenBank. We focused on samples from northeastern Iran (at least one for each haplotype), as they are geographically close to the putative range of *G. s. gracilicornis*, and additionally included samples belonging to each of the frequently found haplotypes of cyt *b*, in order to capture the most common haplotypes of D‐loop in *G. subgutturosa* in Iran. We sequenced the complete D‐loop region using the primers and protocol from Sorokin et al. (2011). The new sequences were edited with SeqScape v.2.6 (Applied Biosystems) and aligned with 182 available *G. subgutturosa* sequences (see accession numbers in Table S2) including Iran (Sorkhabad PA, Zachos et al., 2010), Azerbaijan (Shirvan Steppe Reserve, Sorokin et al., 2011), Turkmenistan (Badkhyz Reserve and its introduced individuals to Ogurchinskii Island, Sorokin et al., 2011), Uzbekistan (Bukhara Breeding Center, Sorokin et al., 2011), China (XUAR, Abduriyim, Zibibulla, et al., 2018), and other sequences from GenBank using the Clustal W algorithm (Thompson et al., 1994) implemented in Mega v.5 (Tamura et al., 2011). Of 76 sequences from Uzbekistan, nine (HQ615654‐HQ615662, from Jeyran Ecocenter of Uzbekistan) were considered to belong to the Turkmenistan group because they stem from founder animals from Turkmenistan (Sorokin et al., 2011). For subsequent analyses, the alignment was trimmed to 478 bp as many available sequences were incomplete.

For the D‐loop dataset, the best‐fitting model of DNA substitution was HKY + I+Γ, chosen among 24 models, using the BIC in jModelTest v.2.1.5 (Darriba, Tab oada, Doallo, & Posada, 2012). Phylogenetic analysis was carried out similarly to the analysis of cyt *b* sequences (see above). A MJ network was also constructed using PopART v.1.7 (Leigh & Bryant, 2015) with the default settings.

## 3 RESULTS

### 3.1 Haplotype network of cyt *b*

The new samples from Iran yielded 19 unique new haplotypes of *G. subgutturosa* that are different from the previously published haplotypes from China (H1‐H46 haplotypes, representing 215 individuals—Abduriyim, Nabi, et al., 2018; Dong et al., 2016), Mongolia (H47, KU560652), and the three samples with unknown origin (H48; JN632644, AF036282, and NC020710). In total, we included 67 different cyt *b* haplotypes for *G. subgutturosa* with 85 polymorphic sites throughout its geographic range (Table 1).

**Figure 3:**
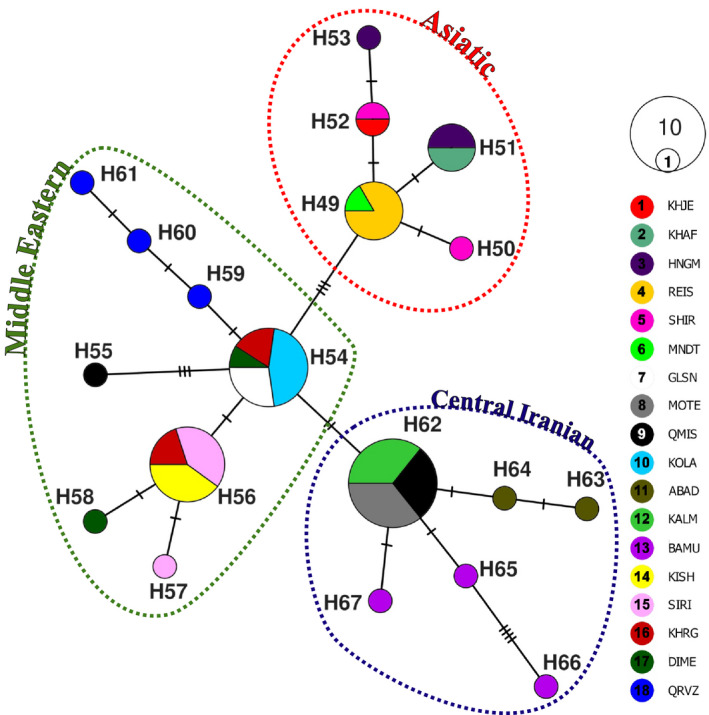
Median‐joining network based on the complete cyt *b* gene depicting the relationships among the three clades described for *G. subgutturosa* in Iran (Asiatic, Central Iranian, and Middle Eastern), delimited by dashed lines. Circle sizes are proportional to haplotype frequencies; colors refer to sampling areas using numbers and abbreviations (compare Figure 2)

The reconstructed MJ network based on an 1,140 bp fragment of cyt *b* provides an overview of the haplotype distribution and relationships within *G. subgutturosa* from Iran (19 haplotypes—Alignment 1, Figure 3). While 13 haplotypes (H50, H53, H55, H57‐H61, and H63‐67) were represented by a single sample, all others were found in at least two specimens. The haplotypes fall into three clades that show a geographic pattern: A Middle Eastern clade (green line), a Central Iranian clade (blue line), and an Asiatic clade (red line—Figure 3). In the Middle Eastern clade (H54‐H61), H54 was the most frequent haplotype occurring in 11 individuals. This group was found to be distributed west of the Zagros Mountains, but H54 and H55 were also detected in Central Iran, and H54 even in one location in northern Iran, east of the Caspian Sea (Golestan NP, Figure 2). In the Central Iranian clade (H62‐H67), H62 was the most frequent haplotype, occurring in 14 individuals. This clade is distributed east of the Zagros Mountains on the Central Iranian Plateau. The most frequent haplotype in the Asiatic clade (H49‐H53) in northeastern Iran was H49 that was found to be present in six individuals. All previously published sequences of *G. subgutturosa* fall within this Asiatic clade when analyzed together with the new Iranian sequences (Alignment 2, Figure 1).

When looking at the short segment (390 bp) of cyt *b* (Alignment 3), three additional haplotypes were found by Khosravi et al. (2019) in Central Iran, while one previously published short haplotype is identical with H54 of the complete cyt *b*, and another is identical with H55. In total, only 29 haplotypes can be distinguished based on the short fragment, so we will not discuss these results further.

### 3.2 Phylogenetic analysis of cyt *b*

All *G. subgutturosa* sequences form a monophyletic clade (posterior probability (PP) = 1; Figure 5) which is placed as sister species to *G. bennettii* (PP = 1). Of the three clades found in the haplotype network, two were monophyletic in the phylogenetic analysis: the Central Iranian clade (blue color, PP = 0.97) and the Asiatic clade (red color, PP = 0.94), including a weakly supported monophyletic group of exclusively Iranian samples (PP = 0.78). The Middle Eastern clade (green color) forms a paraphyletic group at the base of *G. subgutturosa*.

### 3.3 Population differentiation and structure

**Figure 4:**
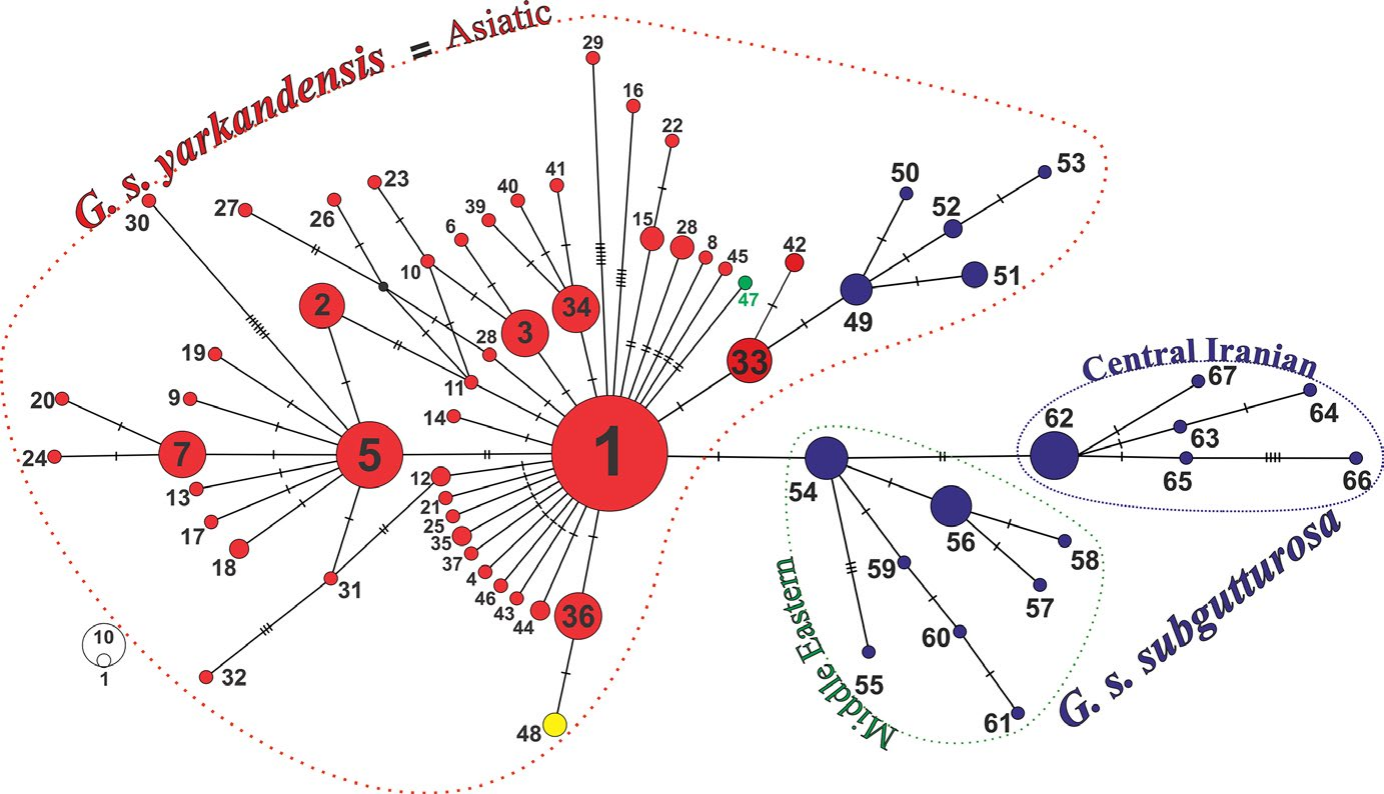
Median‐joining network based on the complete cyt *b* gene depicting the relationships among *G. subgutturosa* in Iran and Asia. Mutational steps among haplotypes are indicated with dashed lines, and small black dots represent inferred missing haplotypes. Each circle represents a different haplotype, whereby areas of circles are proportional to the number of sampled individuals. The color code indicates the origin of the samples (red for China, blue for Iran, green for Mongolia, and yellow for three unknown sequences of GenBank)

In order to obtain an overview of the genetic diversity of *G. subgutturosa*, basic molecular diversity indices were determined for several subgroups of the species, based on geographic patterns (Western group and Eastern group) and phylogenetic clades according to the haplotype network and phylogeny (Table 1). When looking at geographic groups, the Eastern group (China and Mongolia) shows a very similar value for haplotype diversity (*h*: 0.858 ± 0.019) as the Western group (Iran) (*h*: 0.873 ± 0.015), but nucleotide diversity and the mean number of pairwise differences are noticeably larger in the Western group (*π*: 0.00287 ± 0.00017, *k*: 3.3), as compared to the Eastern group **(** *π* : 0.00198 ± 0.00012, *k*: 2.3). When looking at the three clades identified in the haplotype network (Figure 4), the highest level of genetic diversity was found in the Asiatic clade (*h*: 0.877 ± 0.017; *π*: 0.00215 ± 0.00012), while the genetic diversity of the Middle Eastern clade was slightly lower (*h*: 0.715 ± 0.061; *π*: 0.00114 ± 0.00026), and the Central Iranian clade showed the lowest diversity (*h*: 0.468 ± 0.140; *π*: 0.00090 ± 0.00042). When the latter two clades are combined, the diversity indices (*h*: 0.815 ± 0.033; *π*: 0.002 ± 0.00024) are comparable to those of the Asiatic clade. Mismatch distributions were calculated separately for the Asiatic clade, for the combination of Central Iranian and Middle Eastern clades, and for the Total group that includes all sequences (Figure 6). The mismatch distributions for the Asiatic clade and the Total group were unimodal and fully consistent with a population expansion, but the combined Central Iranian and Middle Eastern clades showed a pattern that is interpreted to represent demographic equilibrium. A similar picture is found using *R_2_* statistic (Table 2), which was significant for the Eastern group (*p* < .01) and the Total group (*p* < .01), but not for the Western group. When looking at the clades, only the Asiatic clade shows values indicative of population expansion (*p* < .01). The same picture emerges when looking at Tajima's *D* (Table 2), but here a significant negative value indicative of population expansion was also found for the Central Iranian clade. Fu's *F*_s_ values were highly significant also for the Eastern group, Total group, and Asiatic clade, and much smaller though still significant for the other groups.

### 3.4 Phylogenetic analysis of D‐loop sequences

Eight out of the ten samples were successfully sequenced for D‐loop, including representatives for each northeastern Iranian haplotype (except H53), plus H54, H56, and H62. Two clades are recognized in the analysis of D‐loop sequences (474 bp) in the phylogenetic tree and MJ network (Figures 7 and S1): The first is congruent with the Middle Eastern + Central Iranian clade in the analysis of cyt *b* and comprises sequences from Iran, Azerbaijan, and Turkmenistan. The second clade is congruent with the Asiatic clade in the analysis of cyt *b* and comprises all sequences from China and Uzbekistan plus additional sequences from Turkmenistan and northeastern Iran. Both clades are separated from each other with PP = 0.99. Within the first clade, there are two geographic subclades that show a remarkable amount of acquired mutations: six haplotypes from Azerbaijan form a monophyletic clade (PP = 1) that is separated from the other sequences by at least five mutational steps in the haplotype network; and all six sequences from Turkmenistan form a monophyletic clade (PP = 1) that is separated by at least nine mutational steps.

## 4 DISCUSSION

Our results show that the geography of Iran indeed correlates with subspecies patterning in *G. subgutturosa*, although the main geographic barrier is not the Zagros mountain range, as hypothesized before. Instead, it is the desert areas between Central and northeastern Iran that act as natural barrier, separating two subspecies of *G. subgutturosa*. *G. s. subgutturosa* in Central Iran is connected to the populations in the Middle East and Azerbaijan, while the gazelles in northeastern Iran are more closely related to *G. s. yarkandensis* in Turkmenistan, Uzbekistan, and China.

### 4.1 Geographic patterning and origin of *G. subgutturosa *based on cyt *b*

**Figure 5:**
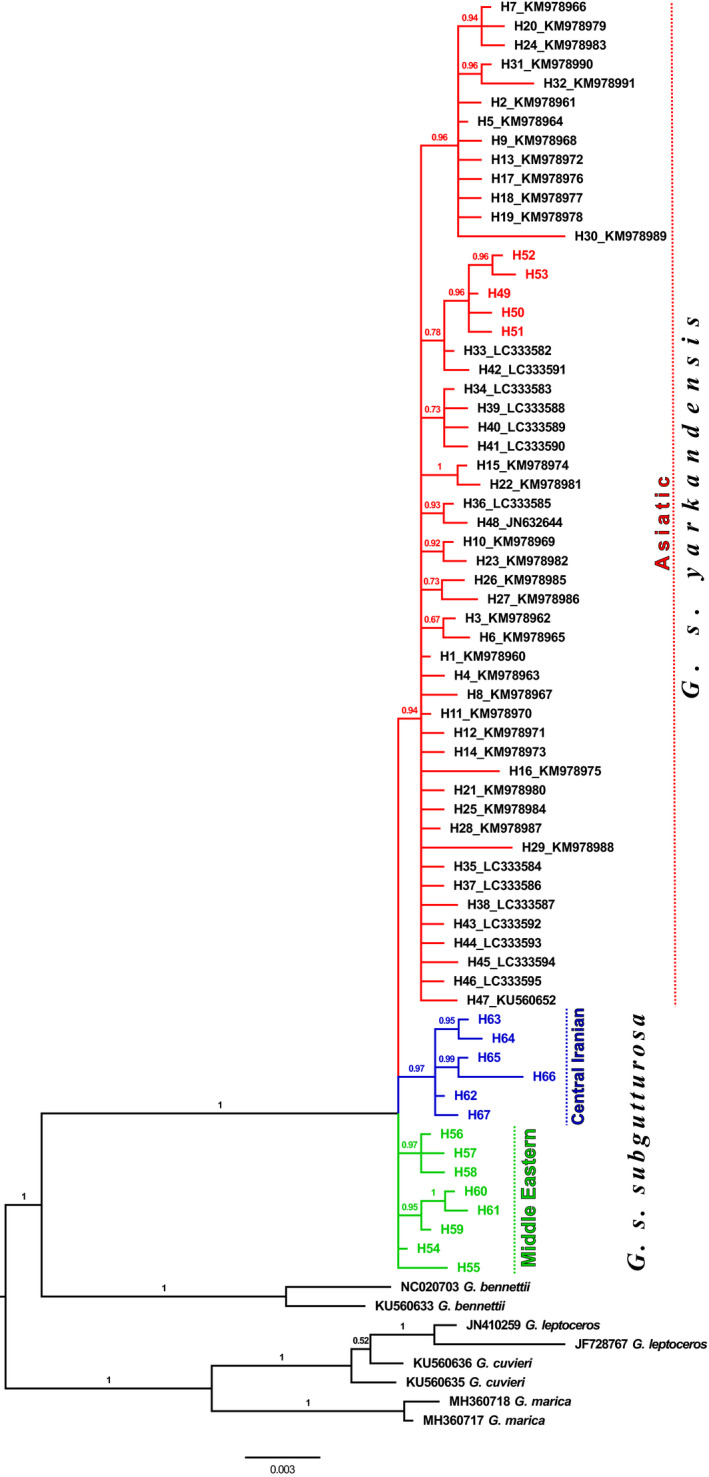
Phylogeny of *G. subgutturosa* from Bayesian analysis of complete cyt *b* sequences. Majority‐rule consensus tree from 15,000 trees sampled from the posterior probability distribution. Iranian samples have colored branches according to the icons in Figure 2. Numbers on nodes are Bayesian posterior probabilities. Each haplotype is only represented by one sequence; labels show GenBank accession numbers

The haplotype networks (Figures 3 and 4) and the phylogenetic tree (Figure 5) are congruent with each other and show a geographic patterning of *G. subgutturosa* samples. Three clades can be identified in the haplotype network, two of which are monophyletic in the phylogeny: an Asiatic clade including samples from Khorasan Province in northeastern Iran, and a Central Iranian clade that is found on the Central Iranian Plateau east of the Zagros mountain range. The third clade, our Middle Eastern clade, is mostly distributed west of the Zagros mountain range and forms a paraphyletic group at the base of *G. subgutturosa* in the phylogeny. Interestingly, the central and most common haplotype of this group (H54) was also found in Central Iran and even in one locality in northeastern Iran. In the haplotype network, both other clades, that is, the Asian and the Central Iranian clade, derive from this haplotype with only one or two mutational steps distance respectively (Figure 3). Furthermore, H54 is the haplotype that is closest to the root of *G. subgutturosa* in the phylogeny, so we propose that it is the ancestral haplotype of the species. Following this interpretation, we argue that *G. subgutturosa* might have originated in the Middle East and expanded from there into Asia. This range expansion is also reflected in the high number of rare alleles in the Asiatic clade that are arranged in a star‐like pattern (Figure 4) with only one or two mutational steps distance around a very frequent central haplotype (Slatkin & Hudson, 1991). Interestingly, Lerp et al. (2016) found the Middle East to be the most likely center of origin for the entire genus *Gazella* with different species emerging there and later colonizing Africa or central Asia. Our study further supports the idea of a gazelle diversity hotspot in an area that ranges from west of the Zagros Mountains to the Mediterranean Sea. This area was strongly affected by the Alpine orogeny (Tchernov, 1988) and especially by the glacial cycles during the Pleistocene when the first ancestors of *G. subgutturosa* might have emerged (Lerp et al., 2016). Rapid habitat changes might have promoted the subsequent range expansion in this group.

### 4.2 Population differentiation and taxonomic implications

When looking at population differentiation measures according to geographic proximity, that is, grouping the samples into a Western group (from Iran) and an Eastern group (from China and Mongolia), the haplotype diversity in both groups is similarly high, but nucleotide diversity is much lower in the Eastern group as compared to the Western group. However, dividing the samples according to the clades in the haplotype network, that is, joining the northeastern Iranian samples and the samples from China and Mongolia into one Asiatic clade, and the samples from western and Central Iran into another clade seems to make more sense biologically. We interpret the distinction of these two groups as subspecies patterning and conclude that the Asiatic clade represents *G. subgutturosa yarkandensis*, while the Middle Eastern and Central Iranian clades represent *G. subgutturosa subgutturosa*. The values for genetic diversity of these two subspecies are very similar (*G. s. yarkandensis* = *h*: 0.877 ± 0.017, *π*: 0.00215 ± 0.00012, *k*: 2.5; *G. s. subgutturosa* = *h*: 0.815 ± 0.033, *π*: 0.002 ± 0.00024, *k*: 2.3), although *G. s. yarkandensis* has a much larger number of segregating sites (S: 67, compared to S: 18 in *G. s. subgutturosa*) and haplotypes (H = 53, compared to H = 14 in *G. s. subgutturosa*). Especially for *G. s. subgutturosa* the sampling is still limited, as populations from Azerbaijan, Iraq, and Turkey are not included, so we expect that haplotype and nucleotide diversity are currently underestimated for this subspecies.

The presence of a third subspecies, *G. s. gracilicornis*, was evaluated using D‐loop sequences, as only this marker is currently available for samples from Turkmenistan and Uzbekistan where it is supposed to occur. The haplotype network (Figure S1) and the phylogeny (Figure 7) show a clade of unique haplotypes from Turkmenistan that might be interpreted as representing *G. s. gracilicornis*. However, these haplotypes were found in sympatry with other haplotypes that can clearly be assigned to *G. s. yarkandensis* as they are deeply nested within the Chinese haplotypes. So it is possible that Turkmenistan and Uzbekistan were colonized by *G. subgutturosa* several times. The first immigrants stem from the nominate form, *G. s. subgutturosa*, and seem to have been isolated from their conspecifics for some time, therefore evolving the unique haplotypes that were only found in Turkmenistan. However, at a later time, populations in Turkmenistan were in contact and exchanged individuals with populations in northeastern Iran and China, which introduced many haplotypes that belong to the subspecies *G. s. yarkandensis*. Nowadays the populations in Turkmenistan harbor all these different D‐loop haplotypes in sympatry, and it can be assumed that they would form one clade in analyses of nuclear genes. We therefore conclude that based on mitochondrial genes there is no evidence for the subspecies *G. s. gracilicornis* occurring in Turkmenistan and Uzbekistan, and that these populations belong to *G. s. yarkandensis*. Contrary to Abduriyim, Zibibulla, et al. (2018), we can find no evidence for the presence of three subspecies of *G. subgutturosa* within China.

### 4.3 Population expansion

**Figure 6:**
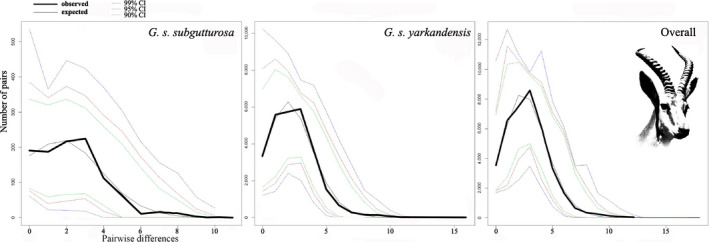
Mismatch distributions of pairwise differences of cyt *b* haplotypes for the *G. s. subgutturosa* from Iran, *G. s. yarkandensis* from northeastern Iran and Central Asia, and for the whole species. Depicted are observed (solid black lines) and expected (solid gray lines) frequencies obtained under a model allowing for demographic expansion

The mismatch distribution analysis clearly supports a demographic expansion for the whole *G. subgutturosa* species, and particularly for the *G. s. yarkandensis* subspecies (Figure 6). For *G. s. subgutturosa* the picture is not very clear, as the curve is relatively flat and does not show a marked peak.

*R_2_* statistics and Tajima's *D* support this interpretation (Table 2). In both statistics, the species as a whole shows clear signs of range expansion, and this expansion can mostly be attributed to the Asiatic clade (*G. s. yarkandensis*). The Middle Eastern clade shows no sign of range expansion in both statistical tests, while the Central Iranian clade has significant values (*p* < .01) for Tajima's *D*, but not for *R_2_*. When both Iranian clades are combined (*G. s. subgutturosa*) no signal for range expansion can be found with *R_2_* and Tajima's *D*. Fu's *F*_s_ shows the same general pattern but seems to overestimate the range expansion for the groups that were found nonsignificant in the other tests. This might be explained by the relatively low sample size and the relatively low number of segregating sites, as demonstrated by Ramos‐Onsins and Rozas (2002). It seems that *G. s. subgutturosa* has not significantly increased its range compared to the historic distribution and still occurs in the geographic area where it originated. This picture might change when sequences from Azerbaijan are included, as it has been shown that the population in Shirvan Steppe Reserve inherits several unique haplotypes for mitochondrial control region sequences compared with sequences from Iran (Sorokin et al., 2011, Figure S1).

### 4.4 Geographic barriers

Iran is a country with very diverse landscapes. The Zagros mountain range spans the country from northwestern Iran to the Strait of Hormuz in the Persian Gulf. In Central Iran, a large plateau exists that is suitable habitat for *G. subgutturosa*, but the mountain range itself is a natural barrier to the distribution of the species and separates the majority of the Middle Eastern clade from the Central Iranian clade. Other natural barriers to the distribution of *G. subgutturosa* are the Dasht‐e Lut and Dasht‐e Kavir deserts in Central Iran and Alborz, Binaloud and Hezar Masjed mountain ranges in the north and northeast of Iran. It seems that the deserts separate the Central Iranian clade from *G. s. yarkandensis* in the northeast (Figures 1 and 2). Crossing these barriers might be feasible for gazelle individuals under certain circumstances, for example, when they are forced to retreat to the plains during heavy snow in extreme winter conditions (Ramezanali Ghaemi, pers. communication). Furthermore, the spreading of *G. subgutturosa* from Iran further into Asia has happened a long time ago, when probably migration was possible due to more favorable climatic conditions at that time.

It has been proposed before that *G. subgutturosa* populations east and west of the Zagros mountain range are separate subspecies (Hayatgheib et al., 2011; Hemami, 1994; Karami et al., 2002). Hayatgheib et al. (2011) compared skulls of animals from both sides of the mountain range and found differences in at least 10 variables. However, the three specimens (two males and one female) that they included from east of the Zagros mountain range were all collected in Khorasan Province in the northeast of Iran. Based on our genetic results (Figures 2 and 3), these specimens belong to the Asiatic clade, that is, to the *G. s. yarkandensis* subspecies, so their results are valid for differentiating *G. s. subgutturosa* from west of the Zagros range and *G. s. yarkandensis* from northeastern Iran, but not from *G. s. subgutturosa* in Central Iran. Morphological differences between *G. s. yarkandensis* from Turkmenistan (Badkhyz Reserve) and Uzbekistan (Bukhara Breeding Center) compared to *G. s. subgutturosa* from Azerbaijan (Shirvan Steppe Reserve) were also reported by Geptner et al. (1961).

Based on our genetic data, *G. s. subgutturosa* occurs on both sides of the Zagros Mountains. One possible reason for perceived morphological differences between the gazelles on the different sides of the mountain range (Karami et al., 2002;Zachos et al., 2010) could be the presence of *G. marica* on the western side. It has recently been shown that mitochondrial haplotypes of *G. marica* occur in wild populations in southwestern Iran in at least two areas (Dimeh PA and Mond PA, Fadakar et al., 2019). In one of these (Dimeh PA), they were found in sympatry with *G. subgutturosa* haplotypes. Based on morphological similarity, individuals from two different source populations were reintroduced to this area, but one of the source populations (Mond PA) might represent a pure *G. marica* population. Therefore, it is possible that cryptic diversity (Pfenninger & Schwenk, 2007; Trontelj & Fišer, 2009) is present west of the Zagros Mountains. In the present study, we found no evidence for *G. marica* east of the Zagros Mountains. Sampling more putative *G. subgutturosa* populations in northern Iraq close to the Iranian border, and other populations west of the Zagros Mountains will help to clarify if *G. marica* is present there. In any case, it would be interesting to morphologically compare individuals belonging to the Middle Eastern clade with those from the Central Iranian clade, to investigate if significant differences can be found, and to incorporate nuclear markers since phylogenetic analyses based on mitochondrial markers are sensitive toward incomplete lineage sorting in closely related species (Wang et al., 2014).

### 4.5 Connectivity of populations

**Figure 7:**
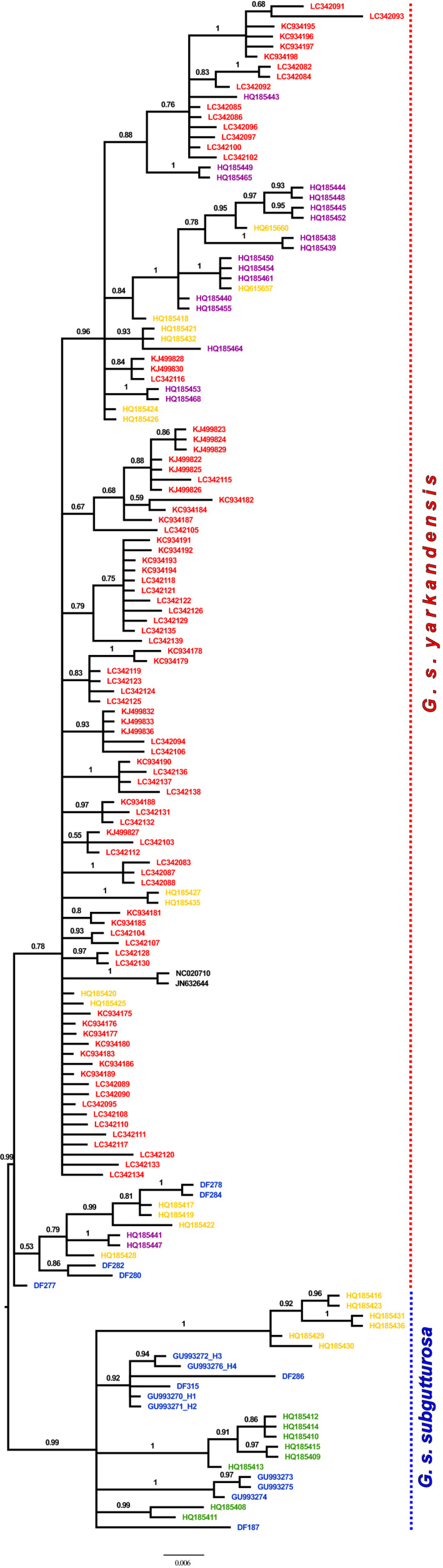
Phylogeny of *G. subgutturosa* from Bayesian analysis of D‐loop sequences from Iran (blue), Azerbaijan (green), Turkmenistan (yellow), Uzbekistan (purple), and China (red). The trees were summarized with the majority‐rule consensus tree. Numbers at nodes represent posterior probabilities

*G. subgutturosa* is, at least in some areas, a migratory species that uses corridors between summer and winter habitats. For example, Fakheran Esfahani and Karami (2005) showed that gazelles migrate from Mooteh WR to Qamishlou NP in winter and back to Mooteh in summer. Within Central Iran, the populations are in relatively close proximity, so exchanging individuals is possible. This is in concordance with the wide distribution of H62 in Central Iran and was also found by Khosravi et al. (2018) based on microsatellite data. The population in Zanjan Province (Sorkhabad PA) in northwestern Iran was also found to be genetically close to gazelles from Shirvan Steppe Reserve in Azerbaijan based on mitochondrial control region sequences (Figure 7, Sorokin et al., 2011). Therefore, it can be assumed that the Central Iranian clade of *G. s. subgutturosa* expands northwards to Azerbaijan. Unfortunately, today *G. subgutturosa* is more and more confined to protected areas (IUCN SSC Antelope Specialist Group, 2017) due to illegal hunting and habitat destruction, so the distances between populations have increased, while more anthropogenic barriers, for example, new roads, led to a reduced connectivity of the remaining populations (Fadakar et al., 2013). It can be expected that the genetic diversity of the Central Iranian *G. s. subgutturosa* will decrease in the next decades if no conservation measures take the connectivity of populations into account (Khosravi et al., 2018).

### 4.6 Implications for conservation

**Figure 8:**
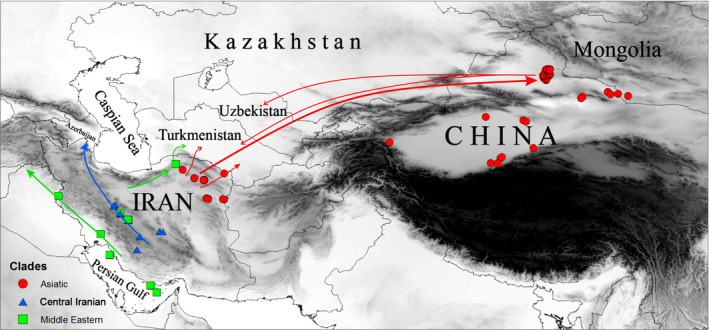
Range expansion of *G. subgutturosa* clades in Asia. The green lines indicate possible range expansions of the Middle Eastern clade west of the Zagros Mountains to Iraq and Turkey; blue lines indicate possible range expansion and connections of populations from Central Iran to west of the Caspian Sea in Trans‐Caucasian habitats, and red lines refer to the expansion of the Asiatic clade from northeastern Iran to China and Mongolia. The shapefile of global country boundaries was downloaded from DIVA‐GIS dataset, and the layout was made in QGIS version 3.10. The gray shade represents topology (hillshade) which was made using the Shuttle Radar Topography Mission (SRTM) elevation model (http://srtm.csi.cgiar.org) in QGIS version 3.10

Based on the cyt *b,* we found three clades of *G. subgutturosa* that occur in Iran, a Middle Eastern clade west of the Zagros mountain range with a putative historical connection to Iraq and Turkey, a Central Iranian clade with a connection to Azerbaijan, and an Asiatic clade in Khorasan Province in the northeast of Iran with connections to Turkmenistan, Uzbekistan, Kazakhstan, Tajikistan, China, and Mongolia (Figure 8). The first two clades are supposed to represent *G. s. subgutturosa*, while the Asiatic clade represents *G. s. yarkandensis*. This is corroborated by morphological differences between the two groups (Geptner et al., 1961; Hayatgheib et al., 2011). We found no evidence to support the existence of a third subspecies, *G. s. gracilicornis*, in Turkmenistan and Uzbekistan based on D‐loop sequences and conclude that gazelles in these areas belong to *G. s. yarkandensis*.

For every future gazelle translocation action, for setting up captive breeding programs, or restocking wild populations, these clades need to be taken into account: Source populations and target populations should belong to the same clade. Most importantly, *G. subgutturosa* from northeastern Iran must not be mixed with the remaining Iranian populations. As pure or hybrid populations of *G. marica* could be present west of the Zagros mountain range, it is especially important to not relocate animals from west of Zagros to Central Iran. As long as the presence of *G. marica* in Iran and Iraq is not studied in detail, conservation efforts for *G. subgutturosa* should focus on Central Iranian populations. The viability of these populations will depend on increased persecution of illegal hunting, inside and outside of protected areas, so that stepping‐stone habitats are maintained and the connectivity of all areas is improved to allow for gazelle migration in a protected area network.

## CONFLICT OF INTEREST

The authors declare that they have no competing interests.

## AUTHOR CONTRIBUTION

**Davoud Fadakar:** Conceptualization (equal); Data curation (lead); Formal analysis (lead); Funding acquisition (equal); Investigation (lead); Methodology (lead); Software (lead); Validation (lead); Visualization (lead); Writing‐original draft (lead); Writing‐review & editing (lead). **Eva Verena Bärmann:** Conceptualization (equal); Data curation (supporting); Formal analysis (equal); Funding acquisition (supporting); Investigation (equal); Methodology (equal); Validation (equal); Visualization (equal); Writing‐original draft (equal); Writing‐review & editing (equal). **Hannes Lerp:** Data curation (supporting); Formal analysis (supporting); Funding acquisition (supporting); Methodology (equal); Validation (supporting); Visualization (supporting); Writing‐original draft (supporting). **Masoumeh Mirzakhah:** Conceptualization (supporting); Data curation (equal); Formal analysis (supporting); Funding acquisition (equal); Investigation (equal); Methodology (supporting); Writing‐original draft (supporting). **Maryam Naseri Nasari:** Conceptualization (supporting); Data curation (equal); Formal analysis (supporting); Funding acquisition (equal); Investigation (equal); Methodology (supporting); Writing‐original draft (supporting). **Hamid Reza Rezaei:** Conceptualization (lead); Data curation (supporting); Formal analysis (equal); Funding acquisition (lead); Investigation (supporting); Methodology (equal); Resources (lead); Software (supporting); Supervision (lead); Validation (equal); Visualization (equal); Writing‐original draft (equal); Writing‐review & editing (supporting). 

## Supporting information

Fig S1

Table S1

Table S2

Supplementary Material

## Data Availability

DNA sequences have been deposited in GenBank under the accession numbers: MT264037‐MT264096.
