# Supplementary material for: Diversification and subspecies patterning of the goitered gazelle (Gazella subgutturosa) in Iran
Source: Ecol Evol. 2020 May 8;10(12):5877–91. doi: 10.1002/ece3.6324 (PMC7319147; doi:10.1002/ece3.6324)
Supplement: Supplementary file 1 — Fig S1 [file ECE3-10-5877-s001.pdf]

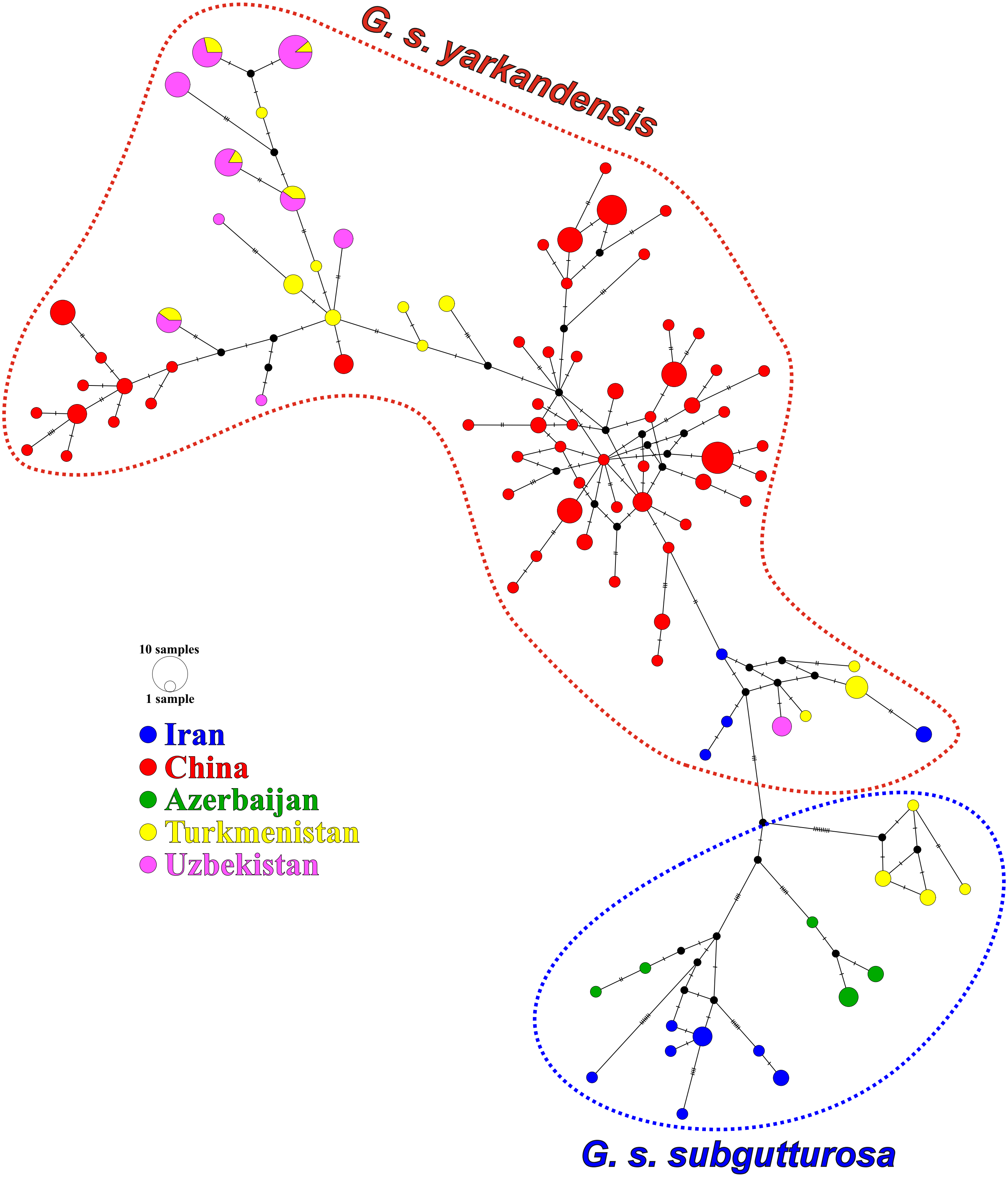
G. s. yarkandensis
G. s. subgutturosa
10 samples
1 sample
Iran
China
Azerbaijan
Turkmenistan
Uzbekistan
