# Supplementary material for: Diversification and subspecies patterning of the goitered gazelle (Gazella subgutturosa) in Iran
Source: Ecol Evol. 2020 May 8;10(12):5877–91. doi: 10.1002/ece3.6324 (PMC7319147; doi:10.1002/ece3.6324)
Supplement: Supplementary file 4 — Supplementary Material [file ECE3-10-5877-s004.docx]

# Supporting Information

Table S1. List of cyt *b* sequences used in this study downloaded from GenBank or obtained by authors of this study.

Table S2. List of D-loop sequences used in this study downloaded from GenBank or obtained by authors of this study.

Figure S1. **Median-joining network based on D-loop gene depicting the relationships among *G. subgutturosa* in Iran and Asia**. Mutational steps among haplotypes are indicated with dashed lines, small black dots represent inferred missing haplotypes. Each circle represents a different haplotype, whereby areas of circles are proportional to the number of sampled individuals.
